# Cost-effectiveness of Dental Workforce Expansion Through the National Health Service Corps and Its Association With Oral Health Outcomes Among US Children

**DOI:** 10.1001/jamahealthforum.2023.0128

**Published:** 2023-03-17

**Authors:** Sung Eun Choi, Ye Shen, Davene R. Wright

**Affiliations:** 1 Department of Oral Health Policy and Epidemiology, Harvard School of Dental Medicine, Boston, Massachusetts; 2 Center for Health Decision Science, Harvard Chan School of Public Health, Boston, Massachusetts; 3 Interfaculty PhD Program in Health Policy, Harvard University, Cambridge, Massachusetts; 4 Department of Population Medicine, Harvard Medical School and Harvard Pilgrim Health Care Institute, Boston, Massachusetts

## Abstract

**Importance:**

Despite considerable efforts to improve oral health for all, large disparities remain among US children. A dental professional shortage is thought to be among the determinants associated with oral health disparities, particularly for those residing in underserved communities.

**Objective:**

To evaluate the cost-effectiveness of expanding the dental workforce through the National Health Service Corps (NHSC) and associations with oral health outcomes among US children.

**Design, Setting, and Participants:**

A cost-effectiveness analysis was conducted to estimate changes in total costs and quality-adjusted life years (QALYs) produced by increasing the NHSC funding for dental practitioners by 5% to 30% during a 10-year period. A microsimulation model of oral health outcomes using a decision analytic framework was constructed based on oral health and dental care utilization data of US children from 0 to 19 years old. Data from the nationally representative National Health and Nutrition Examination Survey (NHANES, 2011-2016) were linked to county-level dentist supply and oral health professional shortage areas (HPSAs) information. Changes in prevalence and cumulative incidence of dental caries were also estimated. Sensitivity analyses were conducted to assess the robustness of results to variation in model input parameters. Data analysis was conducted from August 1, 2021, to November 1, 2022.

**Exposures:**

Expanding dental workforce through the NHSC program.

**Main Outcomes and Measures:**

Changes in total QALYs, costs, and dental caries prevalence and cumulative incidence.

**Results:**

This simulation model informed by NHANES data of 10 780 participants (mean [SD] age, 9.6 [0.1] years; 5326 [48.8%] female; 3337 [weighted percentage, 57.9%] non-Hispanic White individuals) found that when funding for the NHSC program increased by 10%, dental caries prevalence and total number of decayed teeth were estimated to decrease by 0.91 (95% CI, 0.82-1.00) percentage points and by 0.70 (95% CI, 0.62-0.79) million cases, respectively. When funding for the NHSC program increased between 5% and 30%, the estimated decreases in number of decayed teeth ranged from 0.35 (95% CI, 0.27-0.44) to 2.11 (95% CI, 2.03-2.20) million cases, total QALY gains ranged from 75.76 (95% CI, 59.44-92.08) to 450.50 (95% CI, 434.30-466.69) thousand QALYs, and total cost savings ranged from $105.53 (95% CI, $70.14-$140.83) to $508.23 (95% CI, $598.91-$669.22) million among children residing in dental HPSAs from a health care perspective. Benefits of the intervention accrued most substantially among Hispanic children and children in low-income households.

**Conclusions and Relevance:**

This cost-effectiveness analysis using a decision analytic model suggests that expanding the dental workforce through the NHSC program would be associated with cost savings and a reduced risk of dental caries among children living in HPSAs.

## Key Points

**Question:**

Is expanding the dental workforce through the National Health Service Corps (NHSC) program an effective and cost-effective approach to reducing the risk of dental caries among US children in underserved areas?

**Findings:**

This cost-effectiveness analysis using a decision analytic microsimulation model based on data from 10 780 US children found that increasing annual NHSC funding for dental practitioners by 5% to 30% substantially reduced the risk of dental caries and still provided a cost savings. Benefits of the expansion accrued most notably among Hispanic children and children in low-income households.

**Meaning:**

The findings of this cost-effectiveness analysis suggest that expanding the dental workforce through the NHSC would reduce the burden of dental caries among children in underserved areas and address disparities in the social and economic determinants of oral health.

## Teaser

This cost-effective analysis evaluates a proposal to expand the dental workforce through the National Health Service Corps to narrow health disparities and improve dental outcomes among children in the US.

## Introduction

Tooth decay is the most common chronic disease among children in the US, yet oral health is often neglected and may lead to substantial decreases in their quality of life and up to 10 million missed school days annually.^1,2^ Dental care is one of the most important unmet needs in children, with wide disparities affecting minority populations.^3^ US individuals who are Black, Hispanic, have low income, and/or who reside in rural areas are far more likely to have poor oral health compared with non-Hispanic White, high income, urban residents.^4,5^ On average, more than 34 million school hours and $45 billion in productivity are lost annually due to unplanned emergency dental care, mostly among population groups that are underserved and living in vulnerable situations.^6,7^

The interplay of socioeconomic and demographic risk factors, geographic location, dental care utilization, and dental insurance coverage has long-term implications for oral health outcomes and associated costs.^8^ One factor that contributes to oral health disparities is a dental workforce that provides insufficient access to care for the underserved.^9^ Approximately 65.8 million US residents live in a health professional shortage area (HPSA); dental HPSAs are defined by the ratio of dental professionals to the population with high needs, where dental care is in short supply.^10,11,12^ More than 10 600 dental practitioners are currently needed to adequately supply the more than 6300 communities considered to be HPSAs.^10^ In the past, the US Government Accountability Office found shortcomings in the methodology used for designating HPSAs^13^; however, a more recent report by the Office^14^ indicates that the National Health Service Corps (NHSC) is successfully providing care at high-need sites through its scholarships and loan repayment programs whereby students and health professionals receive financial assistance in exchange for serving in an HPSA. Expanding the NHSC program could help to improve access to care for populations living in underserved areas. A prior study observed that NHSC alumni were more likely to work in safety net practices (84% vs 23%) and to treat more publicly insured patients (60% vs 19%).^15^ Evidence suggests that an increased supply of dentists would substantially improve oral health outcomes, including a decrease in the incidence of both tooth decay and bleeding gums.^16^

Research on existing oral health disparities^17,18,19,20,21^ has primarily encompassed observational studies that evaluate how a single clinical or policy initiative has affected disparities in oral health. However, more research is needed to identify the most effective and cost-effective strategies for reducing health disparities and improving the oral health of the population. Given the limited public health resources available, it is critical to identify high-value workforce-related policy interventions that would narrow the disparities and optimize long-term resource allocation. Thus, we sought to fill the gap in the literature by conducting a model-based economic evaluation of whether expanding the dental workforce through the NHSC would advance access to dental care and improve oral health among populations living in dental HPSAs in the US.

## Methods

This study was reviewed and approved by the institutional review board of the Harvard Medical School (IRB21-0885); informed consent was waived because the study used only deidentified data. This study followed the International Society for Pharmacoeconomics and Outcomes Research (ISPOR)^22,23^ reporting guideline.

We constructed and validated a microsimulation model to evaluate how changes in the NHSC program would be expected to affect dental utilization and the risk of the dental caries (tooth decay) based on observational, clinical, and epidemiologic data from the peer-reviewed literature. A microsimulation model using a decision analytic framework was used to account for variations in individual key traits among children residing in dental HPSAs compared with the general population that may critically influence the effectiveness of changes in the NHSC program (eMethods 1 in Supplement 1).

### Data Sources

Table 1 summarizes the key model parameters and data sources^24,25,26,27,28,29,30,31,32,33,34,35^ (additional details are available in eMethods 2-5 and eTables 1-4 in Supplement 1). Demographic, dental utilization, and oral health examination data were obtained from the National Health and Nutrition Examination Survey (NHANES, 2011-2016; N = 10 780 participants <20 years old). This is the only US national survey that collects clinical oral health examination data instead of self-reported dental outcomes, which are often confounded by access to care. To capture characteristics of populations residing in underserved areas, county-level dentist supply and dental HPSA information were merged with NHANES data by the deidentified Federal Information Processing Standard codes linked with NHANES participants at a secure Federal Statistical Research Data Center. Population size and demographic distributions in dental HPSAs and association between dentist supply and oral health outcomes were obtained from this restricted NHANES data and used as model input parameters (eTable 1 in Supplement 1). Survey sample weights were used to correct for differential sampling and nonresponse in the NHANES.^36,37^

**Table 1.** Model Parameters for Estimating Cost-effectiveness of Expanding the Dental Workforce Through the National Health Service Corps

Parameter	Source
Population size of demographic cohorts by urban/rural and dental HPSA status (eTable 1 in Supplement 1)	NHANES 2011-2016 and HRSA
NHSC program details (eMethods 3 in Supplement 1)	HRSA^24^
Disease risk	
Baseline dental caries (eTable 3 in Supplement 1)	NHANES 2011-2016
Baseline dental utilization (eTable 4 in Supplement 1)	NHANES 2011-2016
All-cause mortality rate	CDC^25^
Risk of dental caries (eMethods 2 in Supplement 1)	Model-based estimates
Probability of untreated caries (eMethods 2 in Supplement 1)	Fleming et al, 2018^26^
Probability of tooth abscess for untreated caries (eMethods 2 in Supplement 1)	Monte-Santo et al, 2018^27^
Probability of tooth loss for untreated caries (eMethods 2 in Supplement 1)	Azodo et al, 2012^28^
Effects of dentist supply on dental utilization (eMethods 4 and 5 in Supplement 1)	Heidenreich et al, 2015^29^
Effects of dentist supply on risk of dental caries (eMethods 4 and 5 in Supplement 1)	Guarnizo-Herreno et al., 2014^16^
Disutility weights (eTable 2 in Supplement 1)	Brennan et al, 2004^30^; IHME^31^; Kay et al; 2018^32^
Cost, US $ (eTable 2 in Supplement 1)	Atkins et al. 2016^33^; Humana^34^; and ADA^35^

Abbreviations: ADA, American Dental Association; CDC, US Centers for Disease Control and Prevention; HPSAs, health professional shortage areas; HRSA, Health Resources and Services Administration; IHME, Institute for Health Metrics and Evaluation; NHANES, National Health and Nutrition Examination Survey; NHSC, National Health Services Corps.

### Simulation Model

We simulated a nationally representative sample of 10 000 US residents from 0 to 19 years old starting in 2022 to estimate changes in dental caries incidence with increased NHSC funding for dentist and dental students given differences in disease risks and access to dental care within the dental HPSAs compared with the general US population. The model was simulated for a 10-year period to be consistent with policy planning horizons and to minimize longitudinal uncertainty in the estimates. The simulated individuals were stratified by cohorts defined by age (0-5, 6-12, 13-19 years old); sex; race and ethnicity, by NHANES category (Hispanic [Mexican American and other Hispanic], non-Hispanic Black, and non-Hispanic White); income level, low (<130% of the federal poverty level [FPL]), middle (130%-300% of FPL), and high (>300% of FPL); and residence, by urban or rural and the county with corresponding level of dental professional shortage (whole, partial, or none of the county designated as a dental HPSA).

Dental caries was defined as having signs of decay, being filled on the crown or enamel surface of a tooth, or missing a tooth or teeth owing to caries (with the missing component excluded for primary teeth).^38^ The annual risk of dental caries was estimated for each individual as a function of age, sex, race or ethnicity, and income level (eMethods 2 in Supplement 1). To ensure internal validity, we calibrated the model against dental caries prevalence from NHANES by age groups and race (eFigure 1 in Supplement 1). Dental utilization and incidence rates were updated annually in the simulation model to reflect age and secular trends. When children in the model developed caries, those with untreated caries could develop abscess and tooth loss.^27,28,39^

### Simulated Interventions

**Figure 1.**
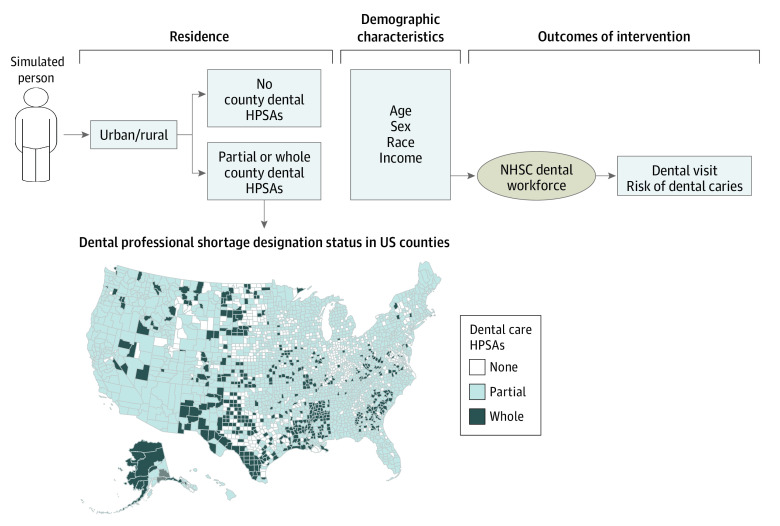
Model Schematic and Dental HPSA Counties in the US Illustration of the simulation model with a map representing county-level dental HPSAs. The map is not generated with the direct outputs of the restricted NHANES data. HPSAs refers to health professional shortage areas; NHANES, the National Health and Nutrition Examination Survey; and NHSC, National Health Service Corps.

During the past 10 years, the number of total NHSC program awards increased by 55%, with more drastic annual increases in recent years (2017-2021) of 14.8% to 25.1%.^40^ Based on these recent changes, we simulated 2 scenarios: (1) status quo (maintaining the status of NHSC program) and (2) base-case scenario with increasing the NHSC funding for dental practitioners by 10% annually (approximately average annual increase in the number of total NHSC awards during the past 5 years; eMethods 3 in Supplement 1). Varying rates of increase were evaluated in the sensitivity analyses. The intervention would affect children residing in dental HPSAs, that is, approximately 14.7 million children (Figure 1).^10^ First, changes in the number of dentists committing to practice in dental HPSAs were estimated based on the current status of the NHSC program, incorporating postservice retention and default rates (eMethods 3 in Supplement 1). Next, expanding the dental workforce in dental HPSAs was estimated to increase dental utilization based on an analysis of Medicaid claims data (with an additional 1 dentist per 10 000 children, the proportion of children utilizing preventive dental care increased by 1.67%),^29^ and changes in the risk of dental caries with increased dentist supply was modeled based on an analysis of national survey data (1 additional dentist per 1000 children reduced the risk of dental caries to an odds ratio of 0.46).^16^ The validity of model parameter assumptions was confirmed with our analysis of restricted NHANES data (details in eMethods 4 in Supplement 1). The estimated outcomes of the simulation interventions included dental caries prevalence, cumulative caries incidence (total number of decayed teeth), and incremental quality-adjusted life years (QALYs) and costs.

### Costs and Utilities

Costs and QALY estimates were integrated during the simulated period for all simulated individuals from a health care perspective. Costs associated with the NHSC program were estimated based on the current number of NHSC awards given to dentists and dental students, and we assumed that this cost was distributed among those in dental HPSAs. Treatment costs were obtained from the American Dental Association claims data and a prior cost-effectiveness analysis (Table 1; eTable 2 in Supplement 1).^33,34,35^ Disutility weights of disease states to calculate QALYs were based on large-scale survey data and prior cost-effectiveness analyses.^30,31,32^ Costs were expressed in 2022 US $ using the Consumer Price Index,^41^ Personal Health Care Dental Service, and Personal Consumption Expenditure,^42^ and costs and QALYs were discounted at 3% annually.

### Sensitivity and Uncertainty Analyses

To assess how variations in the number of awards for dentists and dental students influenced the effectiveness and cost-effectiveness, we varied the annual budget increases from 5% to 30%. Additionally, how length of the dentists’ commitment to serve in dental HPSAs affected outcomes was examined by varying the length of commitment. The current commitment length is 2 years for most of the awards, except for those in the student to service loan repayment program, which requires a 3-year commitment; serving in HPSAs for 1 additional year was assessed.

Additional 1-way sensitivity analyses were performed to investigate changes in the estimated outcomes across a wide range of values for 9 model parameters associated with treatment cost, changes in dental utilization, and risk of developing caries in relation to increased dentist supply, disutility weights, and the NHSC program characteristics (eMethods 4 and 5 in Supplement 1). For these sensitivity analyses, the annual increase of the NHSC funding for dental practitioners was held constant at 10%. Lastly, we performed a probabilistic sensitivity analysis by sampling from the probability distributions of all input parameters (eMethods 5 in Supplement 1). The model was rerun 10 000 times while repeating Monte Carlo sampling from the probability distributions of all input parameters to capture uncertainties in these estimates, generating 95% credible intervals around all outcomes. Supplement 1 provides details of all input data, equations, and complete technical details. Data analyses were performed from August 1, 2021, to November 1, 2022, using R, version 3.6.1 (The R Foundation for Statistical Computing).

## Results

### Model Validation

The simulation population was informed by NHANES data of 10 780 participants (mean [SD] age, 9.6 [0.1] years; 5326 [48.8%] female; 4194 [weighted percentage, 26.4%] Hispanic, 3249 [weighted percentage, 15.7%] non-Hispanic Black, and 3337 [weighted percentage, 57.9%] non-Hispanic White individuals). If there were no changes to the NHSC program and health risk factor profiles, our model estimated that the dental caries prevalence would be 21.3% (95% CI, 18.6%- 24.0%) among 2- to 5-year-olds, 52.1% (95% CI, 48.5%-55.7%) among 6- to12-year-olds, and 56.8% (95% CI, 54.5%-59.1%) among 13- to 19-year-olds (eFigure 1 in Supplement 1). Hispanic children were estimated to have the highest dental caries prevalence, followed by non-Hispanic Black and then, non-Hispanic White children. Additional validation results show that model-predicted values of the status quo matched outcomes from the observed data within less than 5% absolute error (eFigure 1 in Supplement 1).

### Base Case Analysis

**Figure 2.**
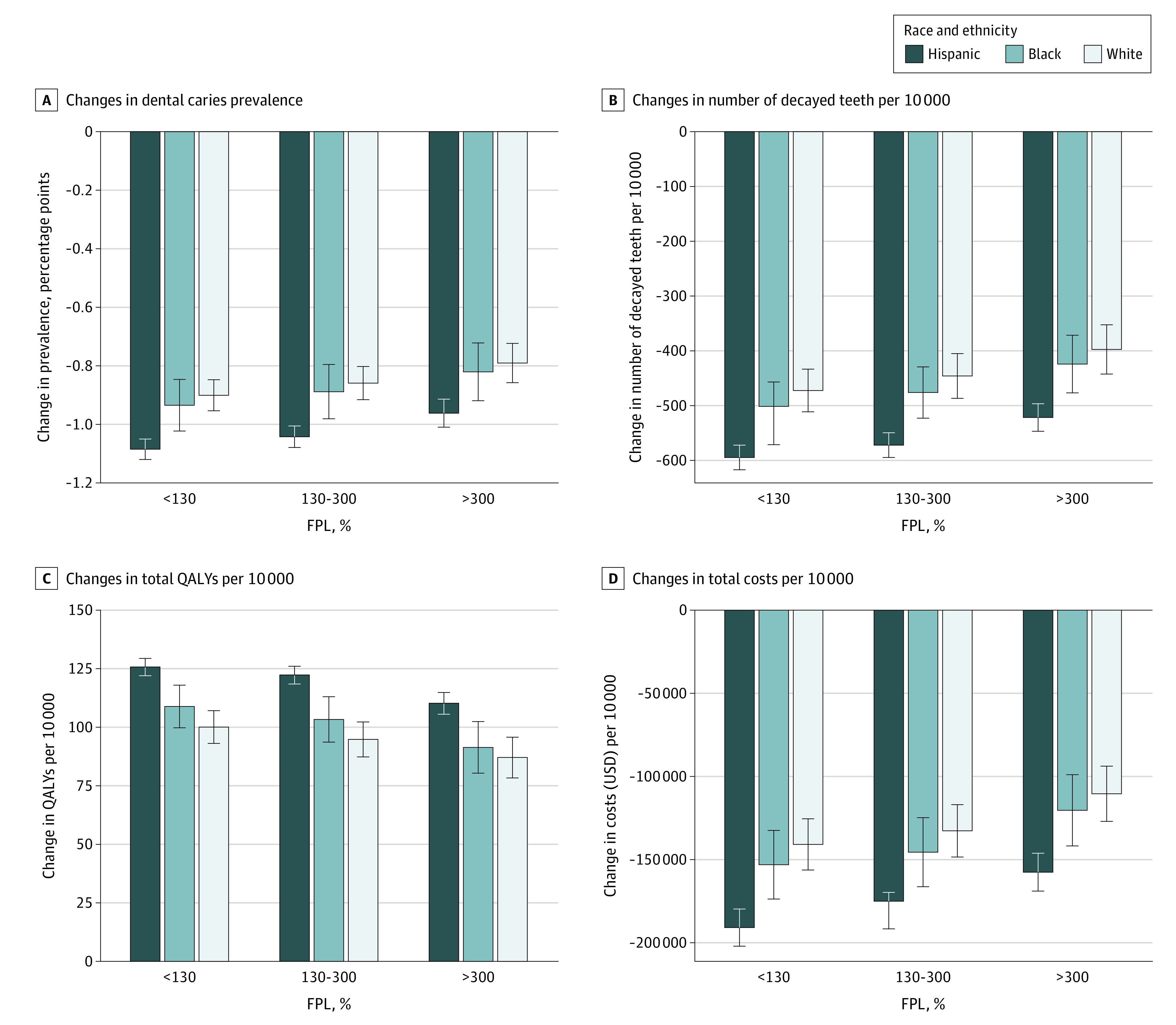
Simulated Outcomes of Increasing the National Health Services Corps Program Budgets by 10% Results were obtained from 10 000 iterations with Monte Carlo sampling, generating 95% credible intervals (whiskers) from the simulation model. These estimates are not direct outputs of the restricted NHANES data. Error bars indicate 95% credible intervals. FPL refers to the federal poverty level, and NHANES to the National Health and Nutrition Examination Survey.

In dental HPSAs, dental caries prevalence among children was estimated to be 56.6% (95% CI, 56.2% to 57.0%) compared with 52.5% (95% CI, 51.6% to 53.4%) in nondesignated areas. Increasing NHSC funding for dental practitioners by 10% while maintaining the length of commitment was estimated to decrease dental caries prevalence by 0.91 (95% CI, −1.00 to −0.82) percentage points, the total number of decayed teeth by 0.70 (95% CI, −0.79 to −0.62) million cases, total costs by $211.39 (95% CI, −$246.73 to −$176.05) million while increasing total QALYs experienced by 150.58 (95% CI, 134.29 to 166.87) thousand QALYs among children residing in dental HPSAs from a health care perspective (Table 2 and Figure 2). When funding for NHSC program increased by 5% to 30%, maintaining the length of dental practitioners’ commitment, the estimated decrease in the total number of decayed teeth was from 0.35 (95% CI, −0.44 to −0.27) to 2.11 (95% CI, −2.20 to −2.03) million cases, total QALY gains were from 75.76 (95% CI, 59.44 to 92.08) to 450.50 (95% CI, 434.30 to 466.69) thousand QALYs, and total cost savings were from $105.53 (95% CI, −$140.93 to −$70.14) to $634.07 (95% CI, −$669.22 to −$598.91) million (Table 2). Dental caries prevalence also decreased by 0.45 (95% CI, −0.54 to −0.36) to 2.72 (95% CI, −2.81 to −2.63) percentage points, corresponding to a 0.6% to 3.7% reduction from the baseline prevalence. Benefits of expansion accrued the most among Hispanic children and children in low-income households residing in dental HPSAs (Figure 2).

**Table 2.** Cost-effectiveness Results and Oral Health Outcomes Among Children Residing in Dental Health Professional Shortage Areas^a^

Scenarios	Change in dental caries prevalence (percentage point)	Change in cumulative dental caries incidence (millions)	Incremental QALYs gained (thousands)	Incremental cost, $ millions
**Percentage increase in budget, with standard length of commitment**				
5	−0.45 (−0.54 to −0.36)	−0.35 (−0.44 to −0.27)	75.76 (59.44 to 92.08)	−105.53 (−140.93 to −70.14)
10	−0.91 (−1.00 to −0.82)	−0.70 (−0.79 to −0.62)	150.58 (134.29 to 166.87)	−211.39 (−246.73 to −176.05)
15	−1.36 (−1.45 to −1.27)	−1.06 (−1.14 to −0.97)	224.77 (208.49 to 241.05)	−317.60 (−352.89 to −282.30)
20	−1.81 (−1.90 to −1.72)	−1.41 (−1.50 to −1.32)	300.05 (283.73 to 316.36)	−423.43 (−458.67 to −388.18)
25	−2.27 (−2.36 to −2.18)	−1.76 (−1.85 to −1.68)	375.63 (359.32 to 391.95)	−529.08 (−564.28 to −493.88)
30	−2.72 (−2.81 to −2.63)	−2.11 (−2.20 to −2.03)	450.50 (434.30 to 466.69)	−634.07 (−669.22 to −598.91)
**Percentage increase in budget, with 1 additional year of commitment**				
0	−0.30 (−0.39 to −0.22)	−0.27 (−0.36 to −0.19)	57.28 (40.95 to 73.62)	−92.92 (−128.27 to −57.56)
5	−0.47 (−0.56 to −0.38)	−0.37 (−0.45 to −0.28)	77.98 (61.65 to 94.31)	−110.37 (−145.72 to −75.01)
10	−0.94 (−1.02 to −0.85)	−0.73 (−0.82 to −0.65)	155.49 (139.15 to 171.82)	−220.98 (−256.29 to −185.67)
15	−1.41 (−1.49 to −1.32)	−1.10 (−1.18 to −1.01)	233.32 (217.04 to 249.60)	−331.39 (−366.69 to −296.10)
20	−1.88 (−1.96 to −1.79)	−1.46 (−1.55 to −1.38)	311.59 (295.28 to 327.91)	−441.29 (−476.54 to −406.04)
25	−2.34 (−2.43 to −2.26)	−1.83 (−1.92 to −1.74)	390.60 (374.28 to 406.91)	−551.59 (−586.79 to −516.39)
30	−2.81 (−2.90 to −2.73)	−2.20 (−2.28 to −2.11)	467.81 (451.62 to 484.01)	−661.61 (−696.76 to −626.45)

Abbreviations: NHANES, National Health and Nutrition Examination Survey; QALY, quality-adjusted life years.

^a^ Results were obtained from 10 000 iterations with Monte Carlo sampling, generating 95% credible intervals from the simulation model. These estimates are not direct outputs of the restricted NHANES data.

When dental practitioners were committed to serve in dental HPSAs for 1 additional year without an increase in the number of awards, dental caries prevalence total number of decayed teeth and costs decreased by 0.30 (95% CI, −0.39 to −0.22) percentage points and 0.27 (95% CI, −0.36 to −0.19) million cases, respectively. Total cost savings and QALY gains were $92.92 (95% CI, −$128.27 to −$57.56) million and 57.28 (95% CI, 40.95 to 73.62) thousand QALYs, respectively. The benefits were also greater when the funding increased from 5% to 30% with an additional year of commitment to serve in HPSA areas by awardees (Table 2).

### Sensitivity Analyses

**Figure 3.**
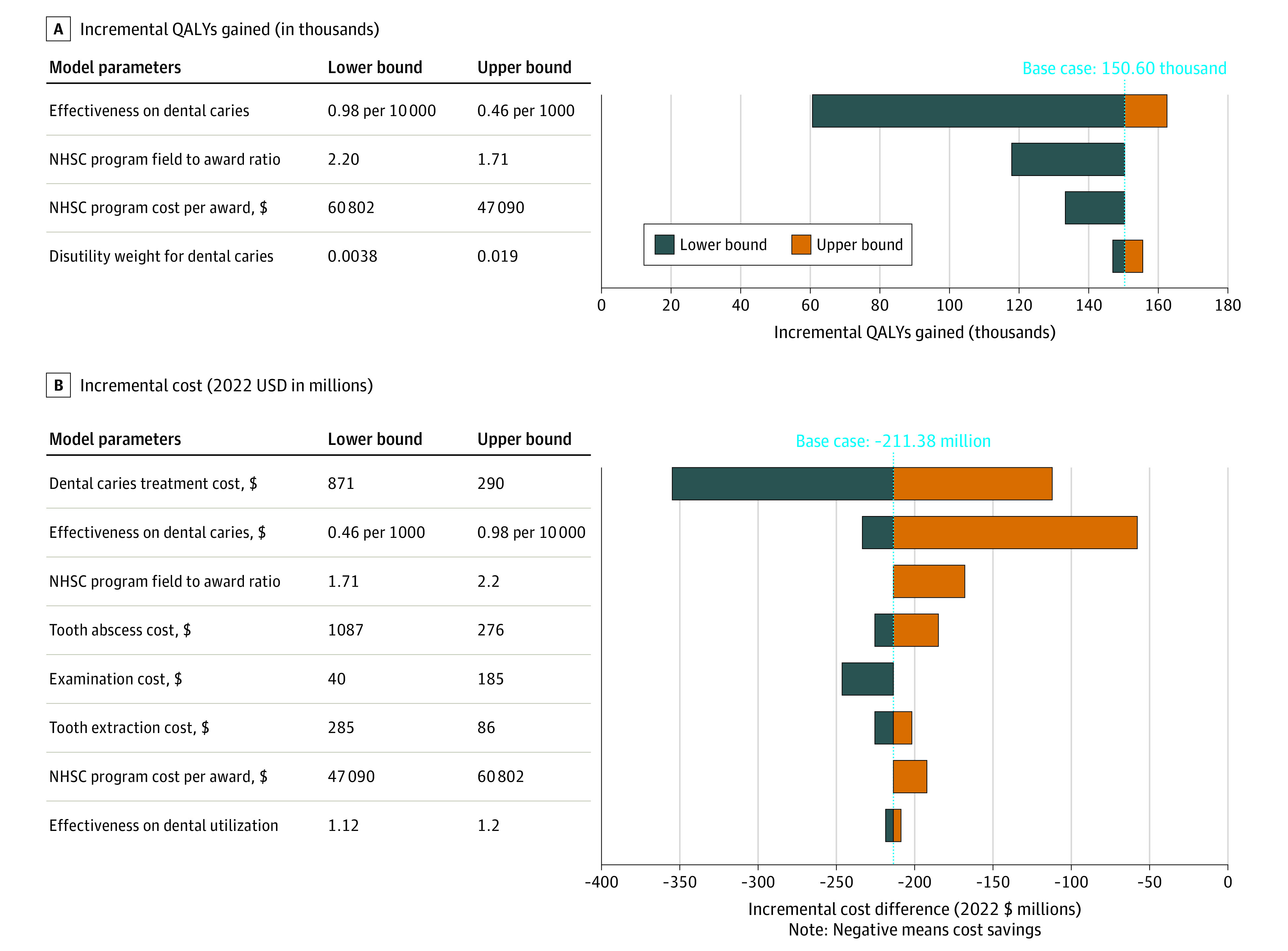
One-way Sensitivity Analysis Results on Incremental QALYs and Cost Results were obtained from 10 000 iterations with Monte Carlo sampling from the simulation model. These estimates are not direct outputs of the restricted NHANES data. NHANES refers to the National Health and Nutrition Examination Survey; NHSC, National Health Services Corps; and QALY, quality-adjusted life years.

No sensitivity analyses substantially changed the fundamental findings. In the 1-way sensitivity analysis (Figure 3), uncertainty around the dental caries treatment cost was the most influential parameter for incremental cost. Uncertainty around the intervention on dental caries was the most influential parameter for incremental QALYs gained and the second most influential for incremental cost. At an odds ratio of 0.98 per 10 000 children for the effectiveness parameter on dental caries, the intervention had the lowest QALY gains of 60.35 thousand QALYs and the lowest cost savings of $56.58 million among all 1-way sensitivity analysis scenarios. In probabilistic sensitivity analyses, expanding the NHSC budget by 10% was the preferred strategy; the probability that expanding the NHSC program was most cost-effective ranged from 90.1% to 99.8% within the willingness-to-pay (WTP) ranges between $0 and $150 000 per QALY (eFigure 2 in Supplement 1).

## Discussion

Expanding NHSC program funding for dentists and dental students would likely have meaningful public health benefits among children residing in dental HPSAs by substantially reducing risk of dental caries, among the most common chronic diseases of childhood in the US. Our findings that expanding NHSC funding would result in cost savings were robust to sensitivity analyses. These estimates are conservative, as not all areas with dental care shortages have HPSA status.^43^ After accounting for variations in disease risks among different demographic groups and demographic composition by dental HPSA status, expanding the NHSC funding would likely benefit low-income Hispanic children the most, a population that disproportionally experiences poor oral health and access to care.^44^

Dental professional shortage in dental HPSAs has been identified as a barrier to access to dental care and gaps in supply and utilization of dental care,^45^ which in turn would affect children’s oral health. The association between dentist supply and use of dental care has been investigated in prior studies using data from the National Survey of Children’s Health and producing mixed results.^44,46,47^ However, the association between dentist supply and clinical oral health outcomes has not been investigated, to our knowledge, except by 1 study that used self-reported oral health outcomes.^16^ In the present study, we assessed the association of dentist supply with clinical oral health outcomes using nationally representative data, then used this association as model input parameters in the simulation model. In addition, via sensitivity analyses, the uncertainty around this model parameter was estimated. Value of information analyses, an approach for assessing whether a strategy should be adopted using currently available evidence, could assess the utility of conducting future observational or experimental studies for informing policy decisions.^48^

In spite of supplemental funding from the American Recovery and Reinvestment Act, the proportion of NHSC oral health professionals did not increase to meet community needs during the same period.^49^ Dentist shortage is projected to continue and increase until at least 2025.^50^ As the dental professional shortage worsens, meeting patient demand will become more difficult to do in a cost-effective manner. A lack of practitioners to serve children living in dental HPSAs may have negative consequences for the oral health of children, highlighting the need for policy initiatives on the supply side, such as higher Medicaid reimbursement rates, direct financial incentives, and/or incorporation of midlevel practitioners.^51,52,53,54,55^ Thus, evaluation of much larger and meaningful long-term implications of dental workforce policy on oral health outcomes and costs, using simulation models, would provide helpful insights for policy makers. Additionally, clinic-based interventions that seek to embed oral health education and treatment into primary care visits are another solution that should be compared head-to-head with more policy-oriented solutions.^56,57,58^ Moreover, future research is warranted to identify the factors associated with the use of dental care on the demand side, such as family demographic information and socioeconomic factors (eg, socioeconomic status, oral health literacy, dental insurance, demand for preventive care).^59,60^

### Limitations

This study had limitations inherent to modeling based on secondary data sources. In the absence of stronger direct evidence, ie, longitudinal observational study assessing how the dental workforce affects oral health outcomes, the association of dentist supply with the risk of dental caries were modeled based on cross-sectional observational studies.^16,29^ Although an association between dentist supply and the risk of dental caries was validated by our analyses of national survey data, the question remains whether the observed association represents a causal relationship. Because there is limited evidence to estimate the effect of the dental workforce on oral health outcomes—accounting for a range of individual and community-level characteristics, including demands for dental care—we assumed a linear relationship between the NHSC expansion and oral health outcomes. Future research should address how dental care utilization behaviors differ among groups in dental HPSAs and what the dose-response relationship between dental workforce expansion and health outcomes is. It is likely that there are complex interactions among location of an individual’s place of residence, accessibility of dental practices, other state-level policies (eg, Medicaid expansion), and treatment seeking behavior, not just the dentist supply. We distributed the dental supply increase across all shortage counties, but policy makers could consider a strategy wherein more disadvantaged areas (defined by HPSA scores) receive more resources.^61^ More data are needed on the population characteristics in unaffected areas to model the potential increase; this is another facet for future research. Next, data from NHANES, which are subject to the limitations of survey studies—recall biases, acceptability biases, underreporting—were used that may have underestimated dental utilization. Because our model estimates the consequences of an intervention on a relative scale to the baseline, this bias would not change the fundamental findings of our study. Finally, although uncertainty analyses were performed by sampling from distributions around the input parameter data sources, all possible uncertainties in a simulation model cannot be captured, hence the results are inevitably subject to the assumptions inherent in decision analytic modeling studies.

## Conclusions

In this cost-effectiveness analysis, the decision analytic model of the proposed policy was associated with a reduced risk of dental caries among children residing in dental HPSAs—those disproportionately at risk of dental caries. Benefits of expanding the dental workforce through the NHSC would likely accumulate among demographic groups who have remained at high risk of dental caries, thereby addressing social and economic determinants and narrowing oral health disparities.

## Notes

Supplement 1. **eMethods 1.** Model simulation **eMethods 2.** Risk of dental caries and associated oral health outcomes **eMethods 3.** National Health Service Corp (NHSC) program and dentist supply **eMethods 4.** Relationship between dentist supply with dental utilization and risk of dental caries **eMethods 5.** Model parameters for one-way or probabilistic sensitivity analysis **eFigure 1.** Model validation for dental caries **eFigure 2.** Cost-effectiveness Acceptability Curve **eTable 1.** Demographic distribution by dental HPSAs and urban/rural status **eTable 2.** Cost and disutility weights **eTable 3.** Baseline prevalence of tooth decay **eTable 4.** Baseline dental utilization

Supplement 2.
Data Sharing Statement
