# Supplementary material for: Cost-effectiveness of Dental Workforce Expansion Through the National Health Service Corps and Its Association With Oral Health Outcomes Among US Children
Source: JAMA Health Forum. 2023 Mar 17;4(3):e230128. doi: 10.1001/jamahealthforum.2023.0128 (PMC10024205; doi:10.1001/jamahealthforum.2023.0128)
Supplement: Supplement 1. — eMethods 1. Model simulation eMethods 2. Risk of dental caries and associated oral health outcomes eMethods 3. National Health Service Corp (NHSC) program and dentist supply eMethods 4. Relationship between dentist supply with dental utilization and risk of dental caries eMethods 5. Model parameters for one-way or probabilistic sensitivity analysis eFigure 1. Model validation for dental caries eFigure 2. Cost-effectiveness Acceptability Curve eTable 1. Demographic distribution by dental HPSAs and urban/rural status eTable 2. Cost and disutility weights eTable 3. Baseline prevalence of tooth decay eTable 4. Baseline dental utilization [file jamahealthforum-e230128-s001.pdf]

Supplemental Online Content

Choi SE, Shen Y, Wright DR. Cost-effectiveness of dental workforce expansion through the National Health Service Corps and its association with oral health outcomes among US children. *JAMA Health Forum.* 2023;4(3):e230128. doi:10.1001/jamahealthforum.2023.0128

eMethods 1. Model simulation

eMethods 2. Risk of dental caries and associated oral health outcomes

eMethods 3. National Health Service Corp (NHSC) program and dentist supply

eMethods 4. Relationship between dentist supply with dental utilization and risk of dental caries

eMethods 5. Model parameters for one-way or probabilistic sensitivity analysis

eFigure 1. Model validation for dental caries

eFigure 2. Cost-effectiveness Acceptability Curve

eTable 1. Demographic distribution by dental HPSAs and urban/rural status

eTable 2. Cost and disutility weights

eTable 3. Baseline prevalence of tooth decay

eTable 4. Baseline dental utilization

This supplemental material has been provided by the authors to give readers additional information about their work.

© 2023 Choi SE et al. *JAMA Health Forum.*

eMethods 1. Model Simulation

We developed a microsimulation model, which simulates dental care use and risk of tooth decay at the level of the individual. The model is stochastic by sampling from probability distributions of input parameters to generate a distribution of outcomes. The model is run in discrete time steps over the life-course from 2022, where the simulated policy changes are introduced at the start of year 202. A model diagram is illustrated in Figure 1.

We classified synthetic population in this model by combinations of a few key demographic characteristics: age (2-5, 6-12, 13-19 years old), sex, race/ethnicity [National Health and Nutrition Examination Survey (NHANES) categories of non-Hispanic white, non-Hispanic black, Hispanic (Mexican-American or other)], and income (relative to the FPL, adjusted for household size), and residing in counties with dental professional shortage (whole, partial, or none of the county designated) within urban/rural regions. These characteristics were obtained from NHANES data linked to dentist supply information obtained from Health Resources and Services Administration (HRSA). Because NHANES is repeated cross-sectional, we had to construct synthetic population to account for the weights. 10,000 individuals were generated, for each cohort defined by the combinations of these characteristics. The model was re-run 10,000 times while repeatedly Monte Carlo sampling from the probability distributions of all input parameters to capture uncertainties in our estimates.[1]

Baseline dental utilization (annual dental visit) and prevalent tooth decay cases were assigned to each simulated individual by repeated Monte Carlo sampling from the probability distributions of each of these variables in NHANES, specific to each demographic group. For dental caries, tooth-level binary indicators for caries incidence were assigned to each simulated individual and

© 2023 Choi SE et al. *JAMA Health Forum.*

summed to calculate the total number of decayed and/or filled teeth for the simulated individuals. To account for individuals aging, we tracked the age of each simulated individual over the simulation period, and updated each individual's probability to utilize dental care and risk of tooth decay to account for their age-specific utilization and health risk by preserving the individual's rank in the population distribution to account for the stability of risk over time.

© 2023 Choi SE et al. *JAMA Health Forum.*

eMethods 2. Risk of dental caries and associated oral health outcomes

Risk of dental caries was modeled as a function of age, race/ethnicity, and income. Below model estimates were adjusted to calibrate to NHANES data (Supplemental Figure S1)

den_caries	Coef.	Linearized Std. Err.	t	P>|t|	[95% Conf.	Interval]
agecat						
6-12	1.77322	.0910509	19.48	0.000	1.590049	1.956391
13-19	1.931936	.1166236	16.57	0.000	1.69732	2.166553
20-29	2.522004	.1328357	18.99	0.000	2.254773	2.789235
racecat						
NH White	-.5726073	.1406452	-4.07	0.000	-.8555489	-.2896656
NH Black	-.1553968	.1546575	-1.00	0.320	-.4665276	.1557339
agecat#racecat						
6-12#NH White	.0465048	.1553039	0.30	0.766	-.2659264	.358936
6-12#NH Black	-.201083	.186286	-1.08	0.286	-.5758422	.1736762
13-19#NH White	.3790678	.1454771	2.61	0.012	.0864057	.6717299
13-19#NH Black	-.1800304	.1741782	-1.03	0.307	-.5304316	.1703709
20-29#NH White	.8320376	.1902897	4.37	0.000	.4492241	1.214851
20-29#NH Black	.1877936	.2219161	0.85	0.402	-.258644	.6342312
incomecat						
130-300% FPL	-.2159971	.0669902	-3.22	0.002	-.3507639	-.0812302
>300% FPL	-.5824465	.0801787	-7.26	0.000	-.7437454	-.4211477
_cons	-1.159482	.0927506	-12.50	0.000	-1.346072	-.9728916

Once individuals develop caries, the probability of caries being treated was 72% based on an analysis of NHANES.[2] For those with untreated caries, the probability of tooth loss was 76.6%,[3] and the probability of tooth abscess was 32.1%.[4]

© 2023 Choi SE et al. *JAMA Health Forum.*

eMethods 3. National Health Service Corp (NHSC) program and dentist supply[5]

The National Health Service Corps (NHSC) supports qualified health care providers dedicated to working in underserved communities in urban, rural, and tribal areas. NHSC-approved sites provide care to individuals regardless of their ability to pay. As of September 30, 2020, there were 7,203 primary care HPSAs, 6,487 dental HPSAs, and 5,733 mental health HPSAs with 16,299 primary care medical, dental, and mental and behavioral health practitioners providing service nationwide in the following programs applied to dental practitioners.

NHSC Scholarship Program (SP): The NHSC SP provides financial support through scholarships, including tuition, other reasonable education expenses, and a monthly living stipend to health professions students committed to providing primary care in underserved communities of greatest need. Upon completion of training, NHSC scholars become salaried employees of NHSC-approved sites in underserved communities. Two years of commitment required.

NHSC Loan Repayment Program (LRP): The NHSC LRP offers fully trained clinicians the opportunity to receive assistance to pay off qualifying educational loans in exchange for service in a HPSA. In exchange for an initial two years of service, loan re-payers receive up to $50,000 in loan repayment assistance. Two years of commitment required.

NHSC Students to Service (S2S) LRP: The NHSC S2S LRP provides loan repayment assistance of up to $120,000 to allopathic and osteopathic medical students and dental students in their last year of school in return for a commitment to provide primary health care in rural and urban HPSAs of greatest need for three years.

State Loan Repayment Program (SLRP): The SLRP is a federal-state partnership grant program that requires a dollar-for-dollar match from the state that enters into loan repayment contracts with clinicians who practice in a HPSA in that state. Two years of commitment required.

NHSC Student Pipeline by Discipline as of 09/30/2020

**Disciplines**	**Students**
Allopathic/Osteopathic Physicians	950
Dentists	315
Nurse Practitioners	78
Physician Assistants	156
Certified Nurse Midwives	28
Total	1,527
**Proportion of dentists among NHSC student pipeline**	**0.206**

NHSC Field Strength by Discipline as of 09/30/2020

**Disciplines**	**Clinicians**
Allopathic/Osteopathic Physicians	2,304
Dentists	1,568

© 2023 Choi SE et al. *JAMA Health Forum.*

Dental Hygienists	428
Nurse Practitioners	3,082
Physician Assistants	1,324
Nurse Midwives	207
Mental and Behavioral Health Professionals	7,173
Other State Loan Repayment Program Clinicians	143
Total	16,229
**Proportion of dentists/dental hygienists among NHSC field strength**	**0.123**

Loan Repayments/Scholarships Awards Table

	Total - FY2020	Dental - FY2020
Loan Repayments	$355,000,000	43,665,000
State Loan Repayments	$15,000,000	1,845,000
Scholarships	$38,000,000	7,828,000
Students to Service Loan Repayment	$20,000,000	4,120,000

*** Dental award costs were estimated based on proportions of dentist/dental students in the field and student pipelines in 2020**

Total NHSC awards table as of 09/30/2020

**Program**	**2015**	**2016**	**2017**	**2018**	**2019**	**2020**	**2021**
Scholarships	196	205	181	222	200	150	149
Scholarship Continuation	11	8	7	7	11	12	12
Loan Repayment	2934	3079	2554	3262	4012	4899	4160
Loan Repayment Continuations	1841	2111	2259	2384	2385	2350	2350
State Loan Repayment	620	634	535	625	812	625	594
Students to Service Loan Repayment	96	92	175	162	127	167	158
**Total Awards**	**5,698**	**6,129**	**5,711**	**6,662**	**7,547**	**8,203**	**7,423**

Total NHSC field strength table as of 09/30/2020

**Program:**	**2015**	**2016**	**2017**	**2018**	**2019**	**2020**	**2021**
Scholars	458	437	405	463	506	573	540
Loan Repayment	8,062	8,593	8,362	8,849	10,221	13,122	13,524

© 2023 Choi SE et al. *JAMA Health Forum.*

Students to Service Loan Repayment	1,136	1,378	179	277	369	388	517
State Loan Repayment	27	85	1,233	1,350	1,957	2,146	1,250
**Total Field Strength**	**9,683**	**10,493**	**10,179**	**10,939**	**13,053**	**16,229**	**15,831**

* Estimated dental NHSC awards and field strength based on proportions of dentist/dental students in the field and student pipelines in 2020.

	Number of awards	Field strength
Scholarship	33	118
Student to service loan repayment	34	80
Loan repayment	801	1614
State repayment	77	264
**Field to award ratio**	2.19	

→ Number of dental awards for each iteration in the simulation model was generated based on the number of dental awards in the past five years (2016-2020) by randomly selecting from a uniform distribution with min: 810 and max: 1170. Field to award ratio in 2020 was used to estimate the number of dentists in the field corresponding the number of dental awards.

Post-service retention rate (Percent of NHSC clinicians retained in service to the underserved for at least one year beyond the completion of their NHSC service commitment) was 80%

Default rate of NHSC Scholarship and Loan Repayment Program participants was ≤ 2.0%

© 2023 Choi SE et al. *JAMA Health Forum.*

eMethods 4. Relationship between dentist supply with dental utilization and risk of dental caries

Author, Year	Study design	Sample size	Study population	Dentist supply unit of analysis	Primary outcome	Findings
Guarnizo-Herreno, 2014[6]	Cross-sectional	63,825	Children aged 1-17 whose mothers completed the National Survey of Children's Health (NSCH) survey	an additional dentist per 1000 population	Odds of tooth decay	OR = 0.46; [95%CI: 0.23, 0.95]
Heidenreich, 2015[7]	Cross-sectional	604,885	Children aged 0-17 enrolled in the Washington State Medicaid Program for ≥11 months	an additional pediatric dentist per 10,000 population	Proportion of Medicaid-enrolled children who utilized preventive dental care	0.0167 percentage point (p=0.047)

In addition to the two published studies above, we tried to validate the findings using restricted NHANES data linked county-level HRSA data on dentist supply. A dataset including NHANES participants for the years 2011-2016 (N=29,919, below table provides population characteristics analyzed) was analyzed to measure the association between dentist supply and 1) annual dental utilization and 2) risk of dental caries.

		Overall (N = 29,919)	Underserved (N = 27,570)	Non-underserved (N = 2,349)
Dentist supply	Dentists per 10,000	6.60 (0.20)	5.51 (0.81)	6.73 (0.23)
Race	Hispanic	8351 (17.1%)	7963 (18.1%)	388 (8.5%)
	Non-Hispanic White	9725 (61.9%)	8571 (60.2%)	1154 (76.8%)
	Non-Hispanic Black	7083 (12.1%)	6660 (12.7%)	423 (7.4%)
	Other	4760 (8.9%)	4367 (9.0%)	384 (7.4%)
Income	Low (< 130% FPL)	10842 (26.8%)	10165 (27.8%)	677 (19.0%)
	Middle (100-300% FPL)	8008 (29.0%)	7387 (29.1%)	621 (27.9%)

© 2023 Choi SE et al. *JAMA Health Forum.*

	High (>300% FPL)	8392 (44.2%)	7473 (43.2%)	919 (53.1%)
Insurance	Private	13162 (59.6%)	11939 (58.7%)	1223 (67.1%)
	Public	10497 (25.0%)	9829 (25.4%)	668 (22.2%)
	Uninsured	4591 (15.38%)	4266 (15.9%)	325 (10.8%)
Education	Less than High School	6873 (17.4%)	6478 (17.8%)	385 (13.1%)
Urban/Rural	Urban	25456 (82.2%)	23753 (83.9%)	1703 (67.4%)

For annual dental utilization, the outcome was a binary indicator for visiting a dental provider in the past 12 months. For the risk of dental caries, the outcome was having any signs of tooth decay (decayed, missing due to caries, or filled teeth). For both outcomes, we estimated logistic regression models, adjusting for all available explanatory variables; age, sex, race/ethnic (Hispanic, non-Hispanic White, non-Hispanic Black), income (<130%, 130-300%, >300 % FPL), insurance type, education attainment (less than high school education), and urban/rural designation.

In the fully adjusted regression models, with an additional dentist per 10,000, annual dental utilization increased with an odds ratio (OR) of 1.29 (95%CI: 1.08, 1.55), and the risk of dental caries decreased with an OR of 0.90 (95%CI: 0.76, 0.98). These results are consistent with the two previously published cross-sectional studies investing the relationship between dentist supply and dental utilization/risk of dental caries. For our base-case parameters, we chose the most conservative parameters; OR of 0.90 (95%CI: 0.76-0.98) per additional dentist per 10,000 for the risk of dental caries and 0.0167 percentage point increase per additional dentist per 10,000 for dental utilization. Supplemental Text S6 provides ranges evaluated in one-way sensitivity analyses.

© 2023 Choi SE et al. *JAMA Health Forum.*

eMethods 5. Model parameters for one-way or probabilistic sensitivity analysis

Variable	Base-case Value	Ranges used in one-way sensitivity analysis	Distribution in case we are doing PSA	Sources
Disutility weights				
Dental caries	0.01	(0.0038 - 0.019)	Beta (20, 1700)	[13-8]
Effectiveness of the intervention				
Dental caries	0.90 per 10,000	(0.46 per 1000- 0.98 per 10000)	Beta (100,5)	[6] and Text S4
Dental Utilization	0.0167 percentage point increase per 10,000	(1.12-1.2) odds ratio per 1,000	Uniform (1.12-1.2)	[14]
Costs				
Examination	185 (10)	(40 - 185)	Gamma (20, 5)	[11]
Dental caries	530 (20)	(290- 871)	Gamma (28, 20)	[10-12]
Abscess	818 (40)	(276 -1087)	Gamma (410, 2)	[15]
Tooth extraction			Gamma (181, 50)	[11]
NHSC program				
Field to Award Ratio	2.20	(1.71- 2.2)	Uniform (1.71, 2.2)	[5]
Program Cost per award	60,802	(47,090- 60,802)	Uniform (47089, 60802)	[5]

Normal distributions were assumed for incidence and mortality model parameters.

Details on construction of ranges used in the sensitivity analysis

For dental caries disutility weight, base-case value is from the Global Burdens of Disease (GBD).[8] The upper bound of the range came from the upper bound of the disability weights in the GBD study and the lower bound came from Kay et al[13]. Kay et al proposed using acute otitis media (a middle ear infection which also involves acute pain and hospital admissions) as an approximation to calculate the impact of tooth decay when it causes pain, due to the lack of utility estimates for the impact of dental caries from the literature. There were three utility estimates for otitis media (OM): 0.72, 0.79, 0.882 in Kay et al[13]. We used the highest utility weight of 0.882 (corresponding to the lowest disutility weight) in Kay et al[13] to calculate the lower bound for the disutility weight for dental caries. The steps to calculate the lower bound for the child with caries is as follows:

- Utility weight of extraction (estimated from OM): 0.882
- Duration of disutility: 12 weeks
- QALY loss for extraction: (1-0. 882)*(12/52) [difference between disutility of decayed and unerupted tooth, multiplied by the time for which pain/extraction impacted]
- Children with caries who experience acute pain: 13.91%
- Mean QALY loss per child with caries: (1-0. 882)*(12/52) *.1391= 0.0038

For the effectiveness of the intervention on dental caries, the lower bound of the range came directly from the point estimate (an odds ratio of 0.46 per 1,000 children aged 1-10 years) in Guarnizo-Herreno et al[6], assuming the same effect on all children in our simulation including those beyond this age group. In the base case, this value was assumed to be only applicable to the same age group and there was no effect among other age groups. The upper bound of the range

© 2023 Choi SE et al. *JAMA Health Forum.*

came from the upper bound of the 95% confidence interval, i.e. 0.90 (95%CI: 0.76, 0.98), in the NHANES analysis.

For the cost of tooth extraction, the lower (upper) bound of the range was calculated by the lowest 10th (the highest 95th) percentile from the national estimates of relevant dental procedure codes for general and pediatric practices in the ADA 2016 survey[11], adjusted for inflation.
In the one-way sensitivity analysis for cost of examination, the lower bound of the range were calculated by the lowest percentile from the national estimates of relevant dental procedure codes for general and pediatric practices in the ADA 2016 survey[11], adjusted for inflation. The upper bound was the base case value as it is higher than the highest 95th percentile estimate form ADA 2016 survey.

For the cost of tooth abscess, the lower (upper) bound of the range was calculated by the lowest (the highest) 2020 quote estimates of relevant dental procedure codes from the United Healthcare Dental Fee Schedule.[15]

For the cost of dental caries, the lower (upper) bound of the range was calculated using the following method:

- Find the lowest 10th (the highest 95th) percentile from the national estimates of dental procedure codes relevant to dental fillings (resin-based composite) for general and pediatric practices in the ADA 2016 survey[11], adjusted for inflation. Do the same for codes relevant to crowns (prefabricated stainless-steel crown).
- Multiply the lowest (highest) numbers from the previous step by Atkins et al[12]'s corresponding estimates for percentages of children with crowns only, with fillings only, or with both crowns and fillings to calculate the lower (upper) bound for the cost of dental caries.

For the two parameters related to NHSC programs, the lower bound of the ranges came from the 2019 values in the HRSA budget justification FY2021 document, adjusted for inflation.

© 2023 Choi SE et al. *JAMA Health Forum.*

eFigure 1. Model internal validation for dental caries

Dental caries (dental caries were calculated based on the number of decayed, missing due to caries, and filled teeth >1). Each colored line represents projected dental caries prevalence from one simulation model iteration. Plots show model outputs from 10,000 iterations with grey shaded area representing the 95% confidence intervals from a Centers for Disease Control and Prevention (CDC) report on dental caries prevalence in the US.

Overall prevalence

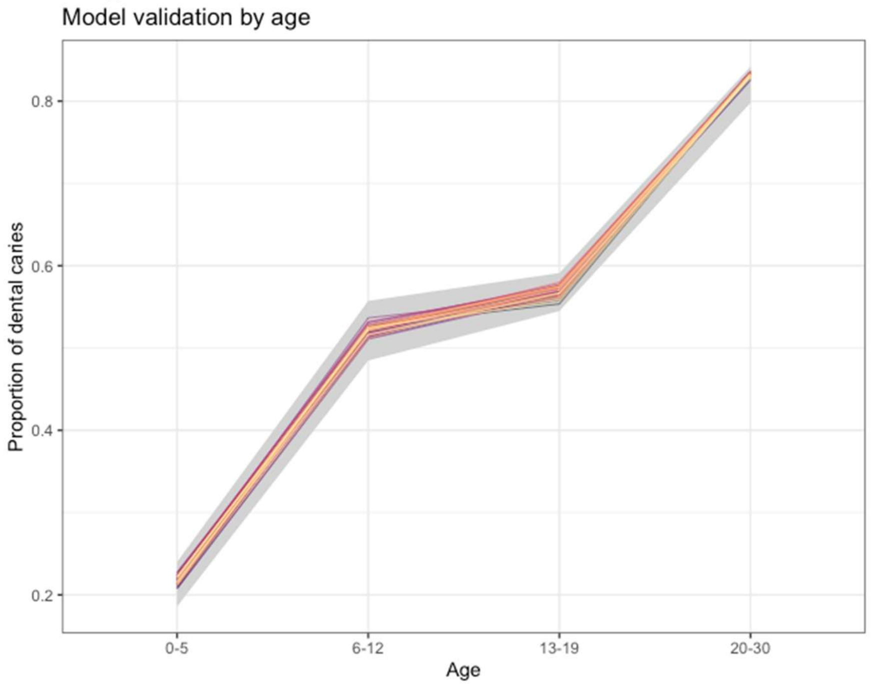

Prevalence by age among Hispanic

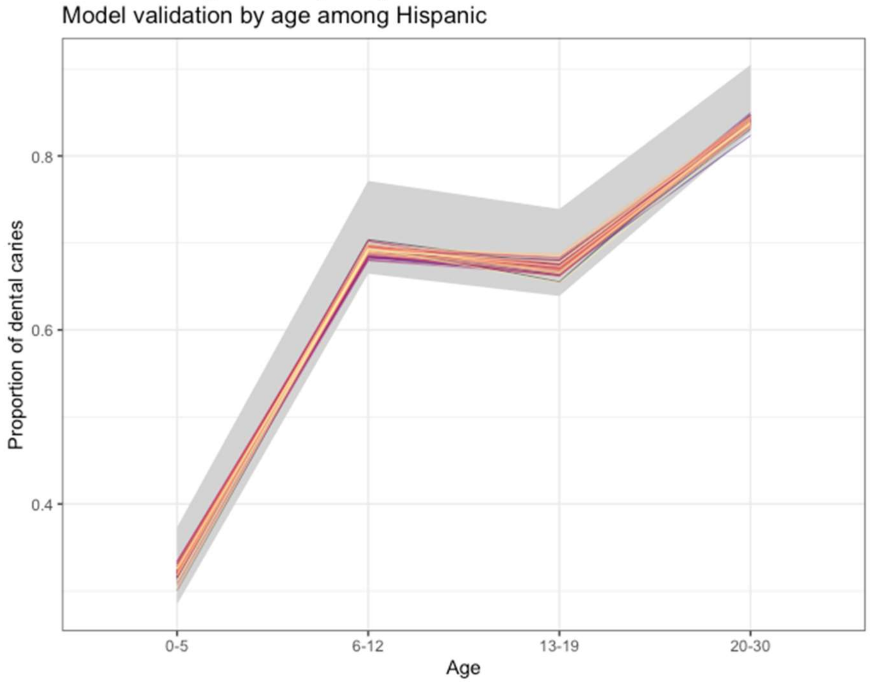

© 2023 Choi SE et al. *JAMA Health Forum.*

Prevalence by age among Non-Hispanic White

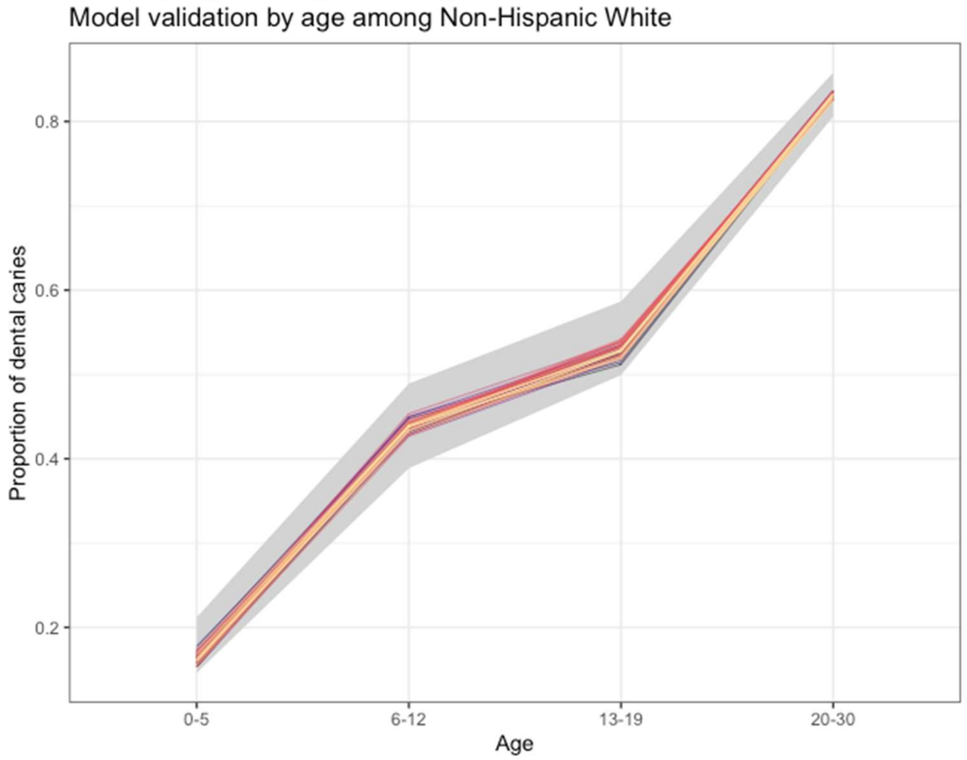

Prevalence by age among Non-Hispanic Black

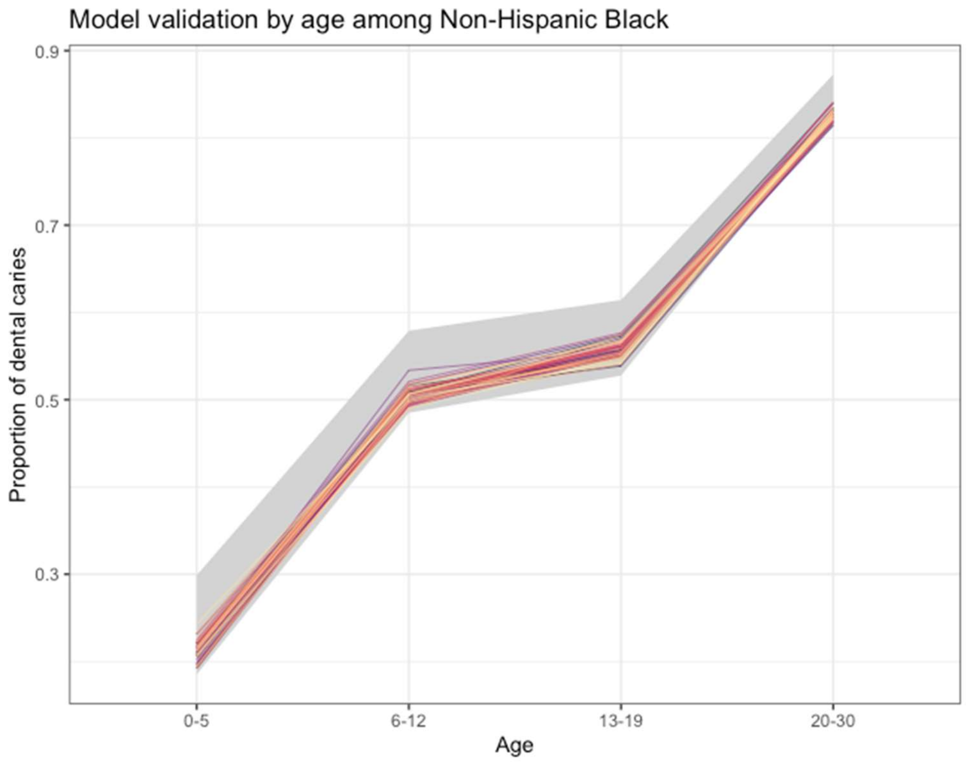

© 2023 Choi SE et al. *JAMA Health Forum.*

eFigure 2. Cost-effectiveness Acceptability Curve

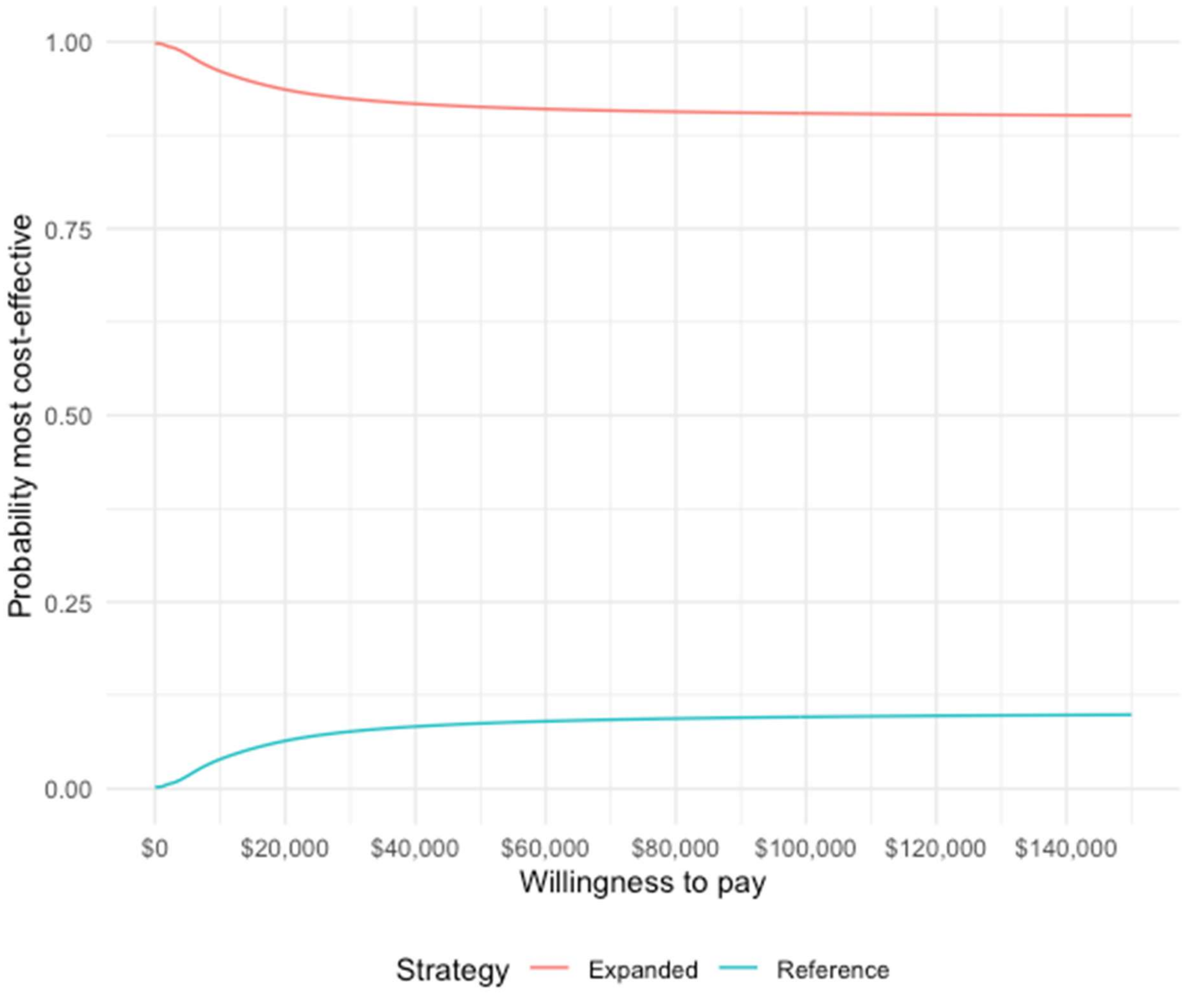

© 2023 Choi SE et al. *JAMA Health Forum.*

eTable 1. Demographic distribution by dental HPSAs and urban/rural status (proportion)

Total number of children residing in dental HPSAs (partial and whole counties in short of supply): 14,688,009

		Urban (81.6%)			Rural (18.4%)		
		Dentist shortage – None (8.34%)	Dentist shortage – Partial (89.79%)	Dentist shortage – Whole (1.87%)	Dentist shortage – None (16.7%)	Dentist shortage – Partial (68.09%)	Dentist shortage – Whole (15.21%)
Age	0 to 5	0.2393	0.2993	0.3514	0.219	0.2724	0.244
	6 to 12	0.3549	0.3541	0.3334	0.3434	0.3618	0.4313
	13 to 19	0.4058	0.3466	0.3151	0.4326	0.3658	0.3248
Sex	Female	0.444	0.4924	0.4206	0.4903	0.4913	0.4682
Race/ ethnicity	Hispanic	0.1486	0.3052	0.6072	0.0883	0.1295	0.2152
	NH White	0.7143	0.506	0.3288	0.8846	0.8206	0.7818
	NH Black	0.1372	0.1888	0.0685	0.0271	0.0499	0.0019
Income	<=130% FPL	0.2518	0.3802	0.4218	0.294	0.3141	0.2424
	130-300% FPL	0.2480	0.2939	0.3832	0.3445	0.3271	0.3502
	>300% FPL	0.5002	0.3259	0.195	0.3615	0.3588	0.4073

These estimates are obtained by accessing restricted de-identified Federal Information Processing Standard (FIPS) identifiers associated with NHANES participants through a Federal Statistical Research Data Center and linked FIPS identifiers to county-level dentist supply and dental care HPSA (none, partial, and whole county designated with dental professional shortage) information.

Disclaimer: The findings and conclusions in this research are those of the authors and do not necessarily represent the views of the Research Data Center, National Center for Health Statistics, or Centers for Disease Control and Prevention.

Data collection for NHANES was approved by the NCHS Research Ethics Review Board. Analysis of de-identified data from the survey is exempt from the federal regulations for the protection of human research participants. Analysis of restricted data through the NCHS Research Data Center is also approved by the NCHS ERB.

© 2023 Choi SE et al. *JAMA Health Forum.*

eTable 2. Cost and disutility weights

Disease states	Disutility weights	Source
Dental caries	0.01	[8]
Tooth extraction	0.073	[8]
Tooth abscess	0.069	[9]

Procedure	Cost (USD)	Source
Examination	185 (10)	[10-12]
Dental caries	530 (20)	[10-12]
Tooth extraction	181 (10)	[10-12]
Tooth abscess	818 (45)	[10-12]

© 2023 Choi SE et al. *JAMA Health Forum.*

eTable 3. Baseline prevalence of tooth decay

			<6	<6	6 to 12	6 to 12	13 to 19	13 to 19
Sex	Race/ethnicity	Income	Mean	SE	Mean	SE	Mean	SE
Male	Hispanic	Low	0.37	0.06	0.80	0.04	0.65	0.07
		Middle	0.16	0.08	0.65	0.09	0.69	0.09
		High	0.11	0.09	0.63	0.13	0.63	0.13
	NH White	Low	0.34	0.07	0.64	0.06	0.63	0.10
		Middle	0.01	0.01	0.55	0.07	0.72	0.09
		High	0.16	0.06	0.45	0.06	0.57	0.06
	NH Black	Low	0.24	0.04	0.61	0.05	0.53	0.06
		Middle	0.14	0.08	0.59	0.08	0.54	0.09
		High	0.39	0.14	0.68	0.10	0.39	0.08
Female	Hispanic	Low	0.28	0.06	0.77	0.05	0.68	0.06
		Middle	0.27	0.09	0.65	0.08	0.76	0.09
		High	0.08	0.06	0.23	0.08	0.65	0.16
	NH White	Low	0.21	0.07	0.51	0.06	0.78	0.06
		Middle	0.09	0.05	0.39	0.08	0.69	0.09
		High	0.07	0.04	0.45	0.07	0.41	0.07
	NH Black	Low	0.32	0.05	0.57	0.06	0.72	0.05
		Middle	0.23	0.10	0.41	0.08	0.56	0.08
		High	0.01	0.01	0.34	0.10	0.54	0.10

*Estimates obtained from NHANES

eTable 4. Baseline Dental Utilization

			<6	<6	6 to 12	6 to 12	13 to 19	13 to 19
Sex	Race/ethnicity	Income	Mean	SE	Mean	SE	Mean	SE
Male	Hispanic	Low	15.65	0.46	40.20	1.84	72.49	2.14
		Middle	13.77	0.76	41.22	2.15	72.21	2.25
		High	14.66	0.77	36.20	3.11	75.35	4.58
	NH White	Low	14.48	0.48	33.71	1.30	73.14	2.36
		Middle	15.01	0.62	37.86	2.08	78.18	4.82
		High	14.80	0.44	36.27	1.23	74.33	2.23
	NH Black	Low	15.36	0.50	35.30	1.25	76.48	2.45
		Middle	14.09	1.35	40.93	2.24	75.23	2.62
		High	15.45	0.90	34.11	2.86	78.35	3.13
Female	Hispanic	Low	14.40	0.38	38.88	1.40	60.63	2.19
		Middle	13.79	0.65	46.19	2.93	65.50	5.13
		High	10.40	0.83	40.34	3.16	59.69	3.65

© 2023 Choi SE et al. *JAMA Health Forum.*

	NH White	Low	14.00	0.43	40.27	2.37	66.85	2.33
		Middle	14.16	0.53	39.27	1.96	64.12	3.89
		High	14.00	0.53	38.09	1.65	65.52	3.37
	NH Black	Low	15.05	0.53	39.06	1.61	70.35	2.41
		Middle	13.30	0.57	40.37	2.10	67.13	2.03
		High	15.28	0.70	43.35	2.30	68.66	4.17

*Estimates obtained from NHANES

© 2023 Choi SE et al. *JAMA Health Forum.*

References

1. Ramsey S, Willke R, Briggs A, et al. Good research practices for cost-effectiveness analysis alongside clinical trials: the ISPOR RCT-CEA Task Force report. *Value Health.* 2005;8(5):521-533.
2. Fleming E, Afful J. Prevalence of Total and Untreated Dental Caries Among Youth: United States, 2015-2016. *NCHS Data Brief.* 2018(307):1-8.
3. Monte-Santo AS, Viana SVC, Moreira KMS, Imparato JCP, Mendes FM, Bonini G. Prevalence of early loss of primary molar and its impact in schoolchildren's quality of life. *Int J Paediatr Dent.* 2018;28(6):595-601.
4. Azodo CC, Chukwumah NM, Ezeja EB. Dentoalveolar abscess among children attending a dental clinic in Nigeria. *Odontostomatol Trop.* 2012;35(139):41-46.
5. Health Resources and Services Administration (HRSA). Justification of Estimates for Appropriations Committees https://www.hrsa.gov/sites/default/files/hrsa/about/budget/budget-justification-fy2022.pdf. Published 2022. Accessed Feb 6, 2022.
6. Guarnizo-Herreno CC, Wehby GL. Dentist supply and children's oral health in the United States. *Am J Public Health.* 2014;104(10):e51-57.
7. Heidenreich JF, Kim AS, Scott JM, Chi DL. Pediatric Dentist Density and Preventive Care Utilization for Medicaid Children. *Pediatr Dent.* 2015;37(4):371-375.
8. Institute for Health Metrics and Evaluation (IHME). Global Burden of Disease Study 2019 (GBD 2019) Disability Weights. https://ghdx.healthdata.org/record/ihme-data/gbd-2019-disability-weights#:~:text=Disability%20weights%2C%20which%20represent%20the,health%20and%201%20equals%20death. Published 2019. Accessed Jan 2, 2022.
9. Brennan DS, Spencer AJ. Disability weights for the burden of oral disease in South Australia. *Popul Health Metr.* 2004;2(1):7.
10. American Dental Association. Survey of Dental Fees. https://success.ada.org/en/practice-management/finances/survey-of-dental-fees. Published 2022. Accessed Feb 13, 2019.
11. Atkins CY, Thomas TK, Lenaker D, Day GM, Hennessy TW, Meltzer MI. Cost-effectiveness of preventing dental caries and full mouth dental reconstructions among Alaska Native children in the Yukon-Kuskokwim delta region of Alaska. *J Public Health Dent.* 2016;76(3):228-240.
12. Humana. Cost of common dental procedures. https://www.humana.com/dental-insurance/dental-resources/cost-of-dental-procedures. Published 2022. Accessed Jan 5, 2022.
13. Kay E, Owen L, Taylor M, Claxton L, Sheppard L. The use of cost-utility analysis for the evaluation of caries prevention: an exploratory case study of two community-based public health interventions in a high-risk population in the UK. *Community Dent Health.* 2018;35(1):30-36.
14. Wehby GL, Shane DM, Joshi A, et al. The Effects of Distance to Dentists and Dentist Supply on Children's Use of Dental Care. *Health Serv Res.* 2017;52(5):1817-1834.
15. UnitedHealthcare. Dental Fee Schedule. https://vaccnprovidertemplate.logisticshealth.com/trainingdocs/VACCN-ProvTrng-DentalFeeSchedule.pdf. Published 2020. Accessed May 15, 2022.

© 2023 Choi SE et al. *JAMA Health Forum.*

© 2023 Choi SE et al. *JAMA Health Forum.*
