# Supplementary material for: Cost-effectiveness of Dental Workforce Expansion Through the National Health Service Corps and Its Association With Oral Health Outcomes Among US Children
Source: JAMA Health Forum. 2023 Mar 17;4(3):e230128. doi: 10.1001/jamahealthforum.2023.0128 (PMC10024205; doi:10.1001/jamahealthforum.2023.0128)
Supplement: Supplement 2. — Data Sharing Statement [file jamahealthforum-e230128-s002.pdf]

Data Sharing Statement

Choi. Cost-Effectiveness of Dental Workforce Expansion Through the National Health Service Corps and Its Association With Oral Health Outcomes Among US Children. *JAMA Health Forum*. Published March 17, 2023. doi:10.1001/jamahealthforum.2023.0128

Data
Data available: No

Additional Information
Explanation for why data not available: Data sharing is not applicable to this study as no new data were collected or created.
